# Investigating the Use of 0.3% Adapalene/2.5% Benzoyl Peroxide Gel for the Management of Moderate-to-Severe Acne in Indian Patients: A Phase 4 Study Assessing Safety and Efficacy

**DOI:** 10.7759/cureus.65894

**Published:** 2024-07-31

**Authors:** Rohit Batra, M Yogendra, Sudarshan Gaurkar, Partha Mukhopadhyay, Dyotona Sen, Sameer Jadhwar

**Affiliations:** 1 Dermatology, Derma World Skin & Hair Clinic, New Delhi, IND; 2 Dermatology, Basaveshwara Medical College, Chitradurga, IND; 3 Dermatology, Government Medical College, Miraj, IND; 4 Dermatology, College of Medicine and Jawaharlal Nehru Memorial (JNM) Hospital, Kalyani, IND; 5 Medical Affairs, Galderma India Pvt. Ltd., Mumbai, IND

**Keywords:** safety and efficacy, india, benzoyl peroxide, adapalene, acne vulgaris

## Abstract

Background

Acne vulgaris is a chronic inflammatory disease of the pilosebaceous unit associated with an increase in sebum secretion. Topical treatment with adapalene and benzoyl peroxide (BPO) is considered effective when used either as monotherapy or in fixed-dose combinations. However, the combination gel of 0.3% adapalene with 2.5% benzoyl peroxide (A0.3%+BPO2.5%) has not been evaluated in Indian patients with acne. This study aimed to evaluate the safety and efficacy of A0.3%+BPO2.5% gel in Indian patients with moderate-to-severe acne vulgaris.

Methodology

This was a 12-week prospective, multicenter, open-label, phase IV study conducted at six centers in India. Safety was assessed based on local tolerability (stinging or burning, erythema, dryness, and scaling) and any reported adverse events. Efficacy was evaluated based on reductions in the number of inflammatory and noninflammatory lesions, the Investigator’s Global Assessment (IGA) scale, and the Global Assessment of Improvement (GAI) score. The patient-reported outcome was measured using the Subject Satisfaction Questionnaire.

Results

Of the 135 patients, 132 completed the study between December 24, 2021, and July 18, 2022 (93.9% had moderate acne; 6.1% had severe acne at baseline). The A0.3%+BPO2.5% gel was well tolerated. The reductions in the severity scores of erythema, scaling, and dryness from baseline to week 12 were 38.9%, 47.4%, and 76.5%, respectively. A targeted reduction of ≥50% in the number of inflammatory and noninflammatory lesions was achieved in 115 (87.1%) and 109 (82.6%) patients, respectively. Based on the investigator’s responses to the IGA questionnaire at week 12, 28% and 40.9% of patients had clear and almost clear skin, respectively. Using the GAI scale, investigators reported that at 12 weeks from baseline, most patients presented with improvements in symptoms, such as erythema, scaling, and dryness, and none reported any worsening. Treatment satisfaction was rated as 91% by the patients.

Conclusions

The A0.3%+BPO2.5% gel effectively reduced the inflammatory and noninflammatory lesions and was found to be safe and well tolerated in Indians with moderate‑to‑severe acne vulgaris.

## Introduction

Acne vulgaris is a chronic inflammatory disorder of the pilosebaceous unit associated with an increase in sebum secretion and is commonly triggered by *Cutibacterium acnes*, one of the anaerobic bacteria that constitute skin flora [1,2]. Although acne vulgaris is common among adolescents, it can affect individuals of various ages. According to the Global Burden of Diseases 2019, acne vulgaris contributed to around 3.52 million disability-adjusted life years in individuals aged 14-59 of both genders [1].

The pathogenesis of acne vulgaris is multifactorial: excessive sebum production, follicular plugging with sebum and keratinocytes, and colonization of hair follicles by *C. acnes*, followed by the release of inflammatory mediators [2]. Factors contributing to acne include excessive exposure to sunlight or the use of specific medications (e.g., lithium, steroids, and anticonvulsants), oil-based cosmetics, facial massages, and occlusive apparel (e.g., shoulder pads and headbands). Endocrine disorders such as polycystic ovarian syndrome and pregnancy, along with genetic factors, repetitive mechanical trauma, increased milk consumption, high glycemic load diets, psychological stress, and insulin resistance may also contribute to the development of acne [2]. Typically, acne develops when sebaceous glands become hypersensitive to normal androgen levels in the blood [2]. Acne presents a diverse range of lesions that can be categorized into the following four grades: grade 1 (open + closed comedones), grade 2 (inflamed lesions + small papules + erythema), grade 3 (pustules), and grade 4 (numerous pustules coalescing into nodules + cysts) [2].

Physical or clinical examinations are usually conducted to diagnose acne vulgaris, although laboratory or radiological tests may be necessary if the clinical assessment suggests underlying hyperandrogenism or other conditions that necessitate additional investigation [2]. According to the American Academy of Dermatology, topical treatments for acne management include retinoids (vitamin A derivatives) such as tretinoin, adapalene, trifarotene, and tazarotene; antibiotics such as erythromycin and clindamycin; and other agents such as benzoyl peroxide (BPO), salicylic acid, niacinamide, azelaic acid, and dapsone. Systemic treatment with a retinoid, such as isotretinoin, is typically reserved for severe cases. Oral antibiotics such as erythromycin and clindamycin are used in moderate-to-severe cases. Hormonal treatment with oral contraceptives and spironolactone is sometimes used to manage the symptoms of premenstrual flare-ups in females [3]. For mild acne, topical therapy alone is often sufficient. However, systemic therapy may be required with topical therapy to treat mild-to-moderate acne [2,4].

Adapalene is a synthetic third-generation topical retinoid that exhibits comedolytic and anticomedogenic properties by normalizing desquamation and blocking inflammatory pathways [5]. BPO, an organic peroxide compound used in acne treatment, functions as a peeling agent. It promotes skin turnover, effectively clearing pores, diminishing the bacterial count (particularly *C. acnes*), and acting directly as an antimicrobial agent [6]. Adapalene is available in various concentrations, including 0.1% and 0.3%, and is often used in once-daily, fixed-dose combinations with 2.5% BPO. In 2001, 2008, and 2020, the Central Drugs Standard Control Organisation (CDSCO) in India approved adapalene 0.1% (A0.1%), A0.1%+BPO2.5% gel, and adapalene 0.3% (A0.3%)+BPO2.5% gel, respectively [7]. Adapalene, being a stable molecule, mitigates concerns about molecular photodegradation, making it suitable for daytime use. Its stability also permits its combination with BPO, resulting in a synergistic effect. This synergy enhances treatment options for moderate-to-severe acne [8]. In a systematic review of 54 clinical trials conducted to assess the efficacy, tolerability, and safety of topical retinoids in treating mild, moderate, or severe acne vulgaris, 0.3% and 0.1% concentrations of adapalene were well tolerated compared with other topical retinoids such as 0.05% tretinoin or 0.1% tazarotene [9]. Currently, as there are no Indian studies on the safety and efficacy of A0.3%+BPO2.5%, the present study was conducted to fill this evidence gap.

## Materials and methods

Study design and patients

A 12-week, prospective, multicenter, open-label, phase IV study (Clinical Trials Registry - India, CTRI/2021/06/034069) was conducted in the following six centers in India: Basaveshwara Medical College & Hospital (Karnataka), Banaras Hindu University (Uttar Pradesh), College of Medicine & Jawaharlal Nehru Memorial (JNM) Hospital (West Bengal), Om Sai Onco-Surgery Multispecialty Centre (Maharashtra), Post Graduate Institute of Medical Science (Chandigarh), and Derma World Skin & Hair Clinic (New Delhi) with IRB numbers ECR/1218/Inst/KA/2019, ECR/526/Inst/UP/2014/RR-17, ECR/674/Inst/WB/2014/RR-17, ECR/1112/Inst/MH/2018, ECR/25/Inst/CH/2013/RR-16, and ECR/310/Indt/DL/2018, respectively. The study was conducted with the approval of CDSCO (following the provisions outlined in the Drugs and Cosmetics Acts and Rules).

Eligibility criteria

Adolescents and adults (12-40 years of age) of either sex with a clinical diagnosis of facial acne vulgaris (moderate or severe) with at least 25-100 inflammatory lesions (papules and pustules) and 30-150 noninflammatory lesions (open and closed comedones) in total (excluding the nose), along with fewer than two acne nodules (≥1 cm), were included. Women of childbearing potential had to have a negative urine test to rule out pregnancy (performed during the baseline visit).

Individuals with acne conglobata, secondary acne (chloracne or drug-induced acne, etc.), nodulocystic acne, acne fulminans, or acne requiring systemic treatment; those with a history of atopic dermatitis, lupus, perioral dermatitis, facial rosacea, or dermatomyositis; those who faced treatment failure with A0.3%+BPO2.5% gel; and those with damage to facial skin (due to cuts, tattoos, skin abrasions, sunburn, or eczema) were excluded from this study. All patients provided written informed consent. The detailed inclusion and exclusion criteria are shown in the Appendices.

Study intervention

The study visit schedule and evaluations at each visit are shown in Table 1.

**Table 1:** Study visit schedule. ^a^: Lesion count (inflammatory and noninflammatory). ^b^: Stinging/burning was not assessed at baseline. ^c^: To be done at the investigator’s discretion. ^d^: Patient diary card review for drug compliance, adverse events, and concomitant medication checks.

Event(s)	Visit 1 – baseline	Visit 2 – week 2 (day 14 ± 1 day)	Visit 3 – week 8 (day 56 ± 3 days)	Visit 4 – week 12 (day 84 ± 3 days)
Informed consent	x			
Demography details	x			
Medical history/Prior therapies	x			
Examination of physical and vital signs	x	x	x	x
Urine pregnancy test	x		x	x
Inclusion and exclusion criteria	x			
Dispensation of investigational product	x		x	
Administration of the investigational product	x	x	x	x
Lesion count^a ^	x	x	x	x
Investigator’s Global Assessment	x			x
Global Assessment of Improvement				x
Local tolerance assessment	x^b^	x	x	x
Photograph^c ^	x			x
Concomitant therapies	x	x	x	x
Adverse events	x	x	x	x
Return of the investigational product			x	
Patient diary card review^d^		x	x	x
Subject Satisfaction Questionnaire				x

The A0.3%+BPO2.5% gel was applied, covering the entire face, once daily for 12 weeks. Each patient received two bottles of A0.3%+BPO2.5% gel (one at baseline and one at week eight). An extra bottle was dispensed only in case the patient lost or destroyed the bottle during the study period. All patients received both verbal and written instructions for proper dosing and treatment application techniques. At the baseline visit, the patients were provided a demonstration of the product application on the face, i.e., four pea-sized dollops to be used to cover the entire face without spot application. Treatment compliance (the number of missed trial product applications) was assessed during diary reviews and interviews on subsequent visits. The importance of following instructions and treatments was emphasized, with instructions to carry the dispensed investigational product and diary for the next visit. Over 12 weeks, patients adhered to a structured procedure, i.e., handwashing, full face cleansing, gentle towel blotting, and the application of a thin gel film, avoiding sensitive areas such as the eyes and mouth. All patients were provided with a moisturizer along with a cleanser to provide symptomatic relief in case of skin dryness/irritation. The use of ancillary products (moisturizer and cleanser) was recorded in the electronic case report form as concomitant therapy. It included all therapies unless specified in the exclusion criteria or prohibited therapies. Vitamin A oral supplements (per recommended daily allowances) and plain penicillin were acceptable. Up to 21 days of systemic anti-inflammatory medication was also deemed acceptable, although this was to be avoided for at least a week before the final assessments.

Safety

Local tolerability was assessed in terms of stinging or burning, erythema, dryness, and scaling, using a four-point scale with scores of 0 (none), 1 (mild), 2 (moderate), and 3 (severe). Any reported adverse events (AEs) were also noted.

Efficacy

Efficacy endpoints comprised mean percentage change in lesion counts, distinguishing between inflammatory (papules + pustules) and noninflammatory lesions (open + closed comedones), evaluated at baseline, week two, week eight, and week 12, and the percentage of patients achieving a minimum of 50% reduction in the number of lesions (inflammatory, noninflammatory, and total lesion counts) by week 12. Investigator’s Global Assessment (IGA) was defined as the severity of facial acne on a scale of 0 (clear) to 4 (severe) at baseline and week 12. The Global Assessment of Improvement (GAI) scale was scored from baseline to week 12 as follows: 5, worsening; 4, no change; 3, minimal improvement; 2, good improvement; 1, very good improvement; 0‍, excellent improvement. Both IGA and GAI were assessed by investigators. Patients were asked to complete the Subject Satisfaction Questionnaire at week 12.

Statistical methods

All statistical tests were performed using the R software. The results are presented as frequencies, percentages, and mean ± standard deviation (SD). Changes in clinical variables between the time points were compared using paired t-tests. Results with p-values <0.05 were considered statistically significant.

## Results

Demographic and clinical characteristics

**Figure 1:**
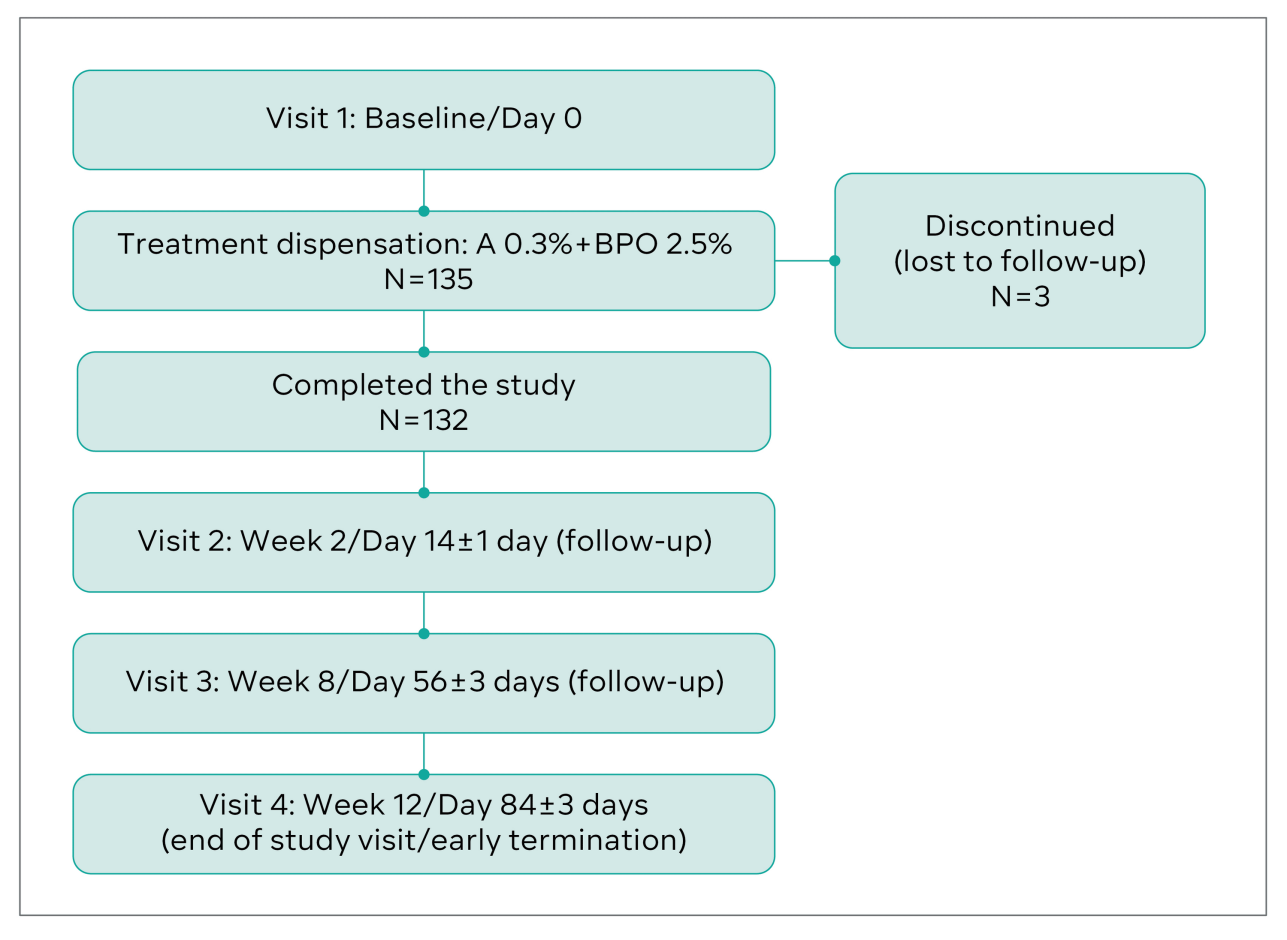
Patient flowchart. A0.3%+BPO2.5% = 0.3% adapalene with 2.5% benzoyl peroxide.

Of the 135 patients enrolled in this study, 132 (97.7%) completed the study between December 2018 and December 2019. The patient flowchart is illustrated in Figure 1. Three (2.3%) patients were lost to follow-up. Of the remaining 132 patients, 78 (59.1%) were female and 54 (40.9%) were male.

Safety

Local Tolerability

Local tolerability (stinging or burning, erythema, dryness, and scaling) results throughout the study are shown in Table 2.

**Table 2:** Percentage distribution of patients based on severity scores from visit 1 to visit 4.

		Baseline	Week 2	Week 8	Week 12
Erythema, n (%)	0—None	70 (53.0)	66 (50.0)	94 (71.2)	89 (67.4)
	1—Mild	53 (40.2)	65 (49.2)	38 (28.8)	43 (32.6)
	2—Moderate	9 (6.8)	1 (0.8)	0	0
	3—Severe	0	0	0	0
Scaling, n (%)	0—None	87 (65.9)	81 (61.4)	107 (81.1)	105 (79.5)
	1—Mild	40 (30.3)	48 (36.4)	25 (18.9)	27 (20.5)
	2—Moderate	5 (3.8)	3 (2.3)	0	0
	3—Severe	0	0	0	0
Dryness, n (%)	0—None	36 (27.3)	88 (66.7)	68 (51.5)	105 (79.5)
	1—Mild	79 (30.3)	14 (10.6)	64 (48.5)	27 (20.5)
	2—Moderate	16 (12.1)	0	0	0
	3—Severe	0	0	0	0
Stinging/Burning, n (%)	0—None	NA	67 (50.8)	89 (67.4)	117 (88.6)
	1—Mild	NA	44 (33.3)	41 (31.1)	13 (9.8)
	2—Moderate	NA	21 (15.9)	2 (1.5)	2 (1.5)
	3—Severe	NA	0	0	0

At baseline (visit one), 53 (40.0%) and nine (6.8%) patients reported mild and moderate erythema, respectively. At 12 weeks, 43 (32.6%) patients reported mild erythema, and none reported moderate erythema. The proportion of patients who reported scaling, dryness, and stinging/burning also decreased from baseline to week 12. None of the patients reported severe stinging or burning, erythema, dryness, or scaling throughout the study.

From baseline to week 12, severity scores for erythema, scaling, and dryness were reduced by 38.9%, 47.4%, and 76.5%, respectively (Table 3).

**Table 3:** Local tolerance scores. NA = not applicable; SD = standard deviation

	Statistics	Baseline	Week 2	Week 8	Week 12
Erythema, n (%)	Mean	0.54	0.51	0.29	0.33
	SD	0.62	0.51	0.45	0.47
	Percent change from baseline	NA	-5.56	-46.30	-38.89
Scaling, n (%)	Mean	0.38	0.41	0.19	0.20
	SD	0.56	0.54	0.39	0.40
	Percent change from baseline	NA	-7.89	-50.00	-47.37
Dryness, n (%)	Mean	0.85	0.88	0.48	0.20
	SD	0.61	0.56	0.50	0.40
	Percent change from baseline	NA	3.53	-43.53	-76.47

Adverse Events

A total of 21 (15.9%) patients reported 23 AEs (22 mild and one moderate). The mild AEs included itching (n = 6), cough and cold (n = 2), cough (n = 2), cold (n = 2), headache (n = 2), fever (n = 4), skin burning (n = 2), toothache (n = 1), and gastric ache (n = 1). One moderate AE reported was loose motion. One patient presented with three AEs (fever, headache, and gastric ache). All AEs were resolved within one to three weeks of their onset.

No serious AEs were documented during the study. All AEs were reported as not related to the investigational product. There were no AEs that led to study discontinuation.

Efficacy

Mean Percentage Change in Lesion Count

From baseline to week 12, the numbers of both inflammatory and noninflammatory lesions were reduced. Table 4 presents the mean inflammatory and noninflammatory lesion counts throughout the study and the mean change from baseline/visit one. At baseline, the mean change in the number of inflammatory lesions was 43.6 (SD = 19.5), and it had reduced to 9.4 (SD = 10.9) by week 12, which was a substantial reduction of 78.3%. Similarly, the mean change in the number of noninflammatory lesion counts was 43.9 (SD = 16.3) at baseline and 9.0 (SD = 8.4) at week 12, which represented a reduction of 79.5%.

**Table 4:** Mean percentage change in inflammatory and noninflammatory lesion counts. NA = not applicable; SD = standard deviation

	Statistics	Baseline	Week 2	Week 8	Week 12
Inflammatory lesion	Mean	43.6	31.0	17.2	9.4
	SD	19.5	17.5	15.6	10.9
	Mean percent change from baseline	NA	-28.8	-60.6	-78.3
Noninflammatory lesion	Mean	43.9	30.8	16.4	9.02
	SD	16.3	13.7	12.0	8.4
	Mean percent change from baseline	NA	-30.0	-62.7	-79.5
Total inflammatory and noninflammatory lesion count	Mean	87.5	61.8	33.6	18.5

A reduction in the number of inflammatory and noninflammatory lesions of ≥50% at week 12 was achieved in 115 (87.1%) and 109 (82.6%) patients, respectively.

Investigator’s Global Assessment Score

**Figure 2:**
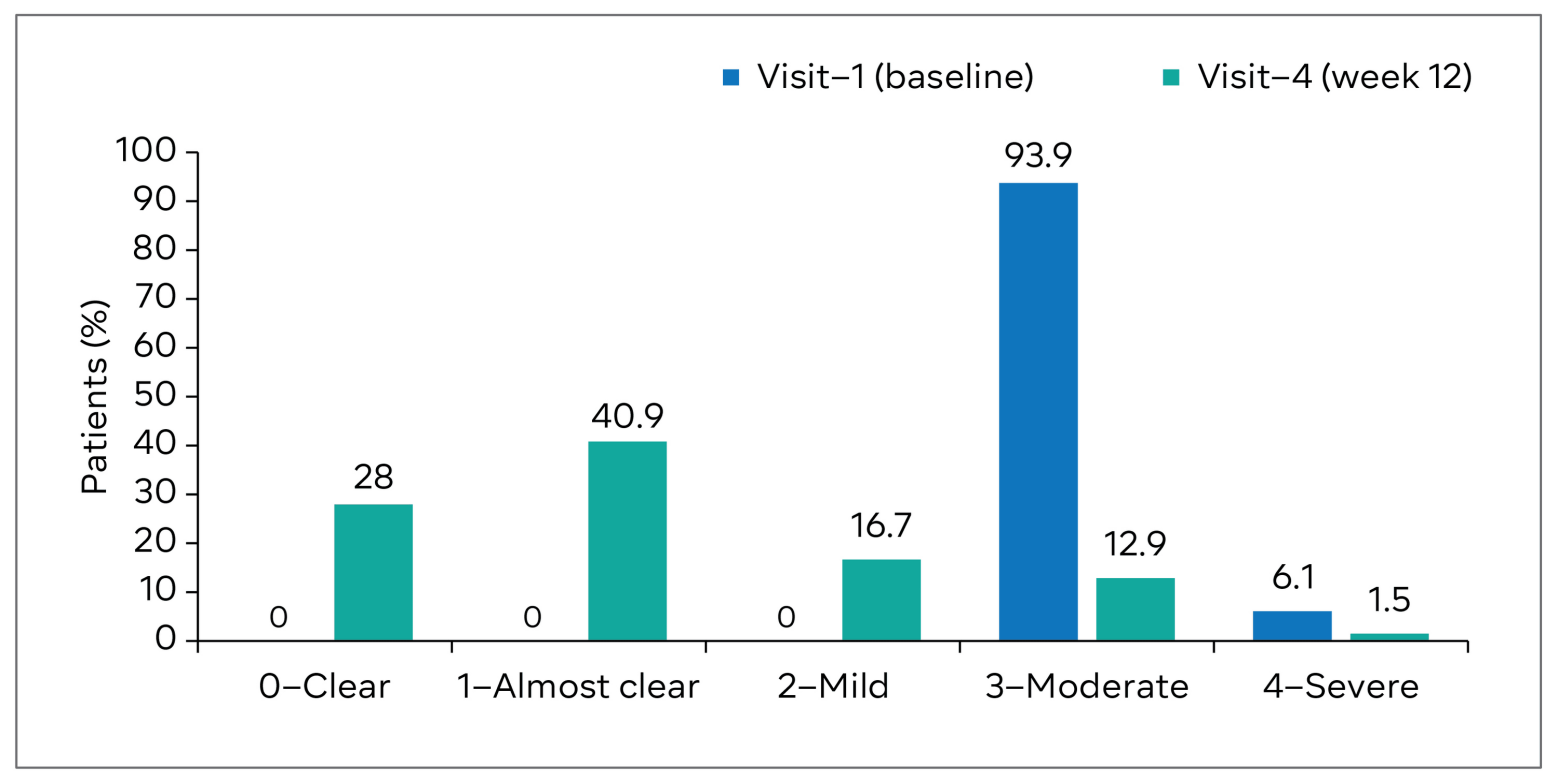
Investigator Global Assessment (IGA) score on acne severity.

Based on the assessment of the IGA score by the investigator, 124 (93.9%) patients had moderate acne and eight (6.1%) had severe acne at baseline. At 12 weeks, 37 (28.0%) and 54 (40.9%) patients had “clear” and “almost clear” skin, respectively (Figure 2).

Global Assessment of Improvement Score

Regarding improvement in the GAI score from baseline at week 12, investigators reported that 34 (25.8%), 51 (38.6%), and 33 (25%) patients experienced excellent, very good, and good improvement, respectively. None of the patients had “no change” or “worsening” symptoms.

Patient-reported outcomes

Regarding treatment satisfaction at week 12, 68 (51.5%) patients were “satisfied,” 52 (39.4%) were “very satisfied,” and 12 (9.1%) were “somewhat satisfied.” None of the patients reported dissatisfaction with the treatment.

## Discussion

The findings from this study support the use of A0.3%+BPO2.5% as an effective acne therapy in both genders in Indian patients (ages 12-40 years) with acne.

Adapalene, a retinoid, reduces dyskeratosis at the pilosebaceous unit, inhibits the formation of microcomedones, and has mild anti-inflammatory properties. BPO is an effective broad-spectrum antibacterial agent that has anti-inflammatory and comedolytic properties. According to the American Academy of Dermatology, adapalene and BPO can be used as monotherapy for mild acne but are often used in fixed-dose combinations to manage moderate‑to-severe acne vulgaris [4]. The topical combination of the retinoid adapalene and the antimicrobial BPO allows for the simultaneous targeting of multiple pathogenic factors associated with acne. Adapalene downregulates the cell surface retinoic acid receptor used by *C. acnes* for the induction of inflammatory cytokine production while BPO eliminates *C. acnes*. Additionally, adapalene modifies the follicular microclimate, thereby enhancing the penetration of BPO. This demonstrates the synergistic action of the adapalene/BPO combination across various clinical settings [10]. Treatment with the combination gel for 12 weeks has been reported to be safe and well tolerated [8,11]. In the study by Dréno et al. [12], mild forms of scaling, dryness, erythema, and stinging/burning were reported with A0.3%+BPO2.5%, especially in the first week. In the present study, erythema, scaling, and dryness were reported throughout the study, but these were mostly mild‑to‑moderate, and the number of patients reporting these symptoms reduced as the study progressed. Stinging/burning was reported only from week two, but 88.6% of patients reported no stinging/burning by week 12.

In a 24-week study (N = 67), skin irritation (14.9% with A0.3%+BPO2.5% vs. 6% with the vehicle) and pain (3.0% with A0.3%+BPO2.5% vs. 1.5% with the vehicle) were reported as common AEs [12]. In another 24-week study, the topical application of the A0.3%+BPO2.5% gel was reported to cause mild AEs, such as dry skin (4.4%) and skin irritation (4.4%) [13]. However, short contact therapy (15-30 minutes) also works well in patients prone to side effects such as irritation or erythema [14,15]. In the present study, the combination gel was reported to cause 23 AEs in 21 (15.9%) patients, with the most common AE being itching (six of 23 AEs, 26.1%), which was reported in six (4.5%) of 132 patients. However, with the continued use of the gel, all tolerability parameters improved at the end of the study. None of the AEs were reported to be related to the treatment. Noncomedogenic moisturizers and pH-balanced cleansers increase hydration and maintain skin barrier integrity when used as an adjunct to adapalene gel, which improves local tolerance and adherence [16,17]. This was the reason for providing patients with moisturizers and cleansers.

In the report by Dréno et al. [12], inflammatory as well as noninflammatory lesions were reduced by 86.7% and 59.5%, respectively, from baseline at week 24. In a comparative, open-label, randomized controlled trial of individuals with mild-to-moderate acne (N = 80), a combination of A0.1%+BPO2.5% resulted in reductions of 63.5% and 29.2% in inflammatory and noninflammatory lesions, respectively [18]. In another study on individuals with mild-to-moderate acne, the total lesion count was reduced by 61.55% after treatment with A0.1%+BPO2.5% for 12 weeks [19]. These studies also showed that A+BPO was better at reducing lesion counts. The higher concentration of adapalene used in the present study, i.e., 0.3% vs. 0.1%, could be attributed to the greater reduction in lesion counts. In another randomized controlled trial, A0.3%+BPO2.5% was reported to reduce both inflammatory (68.7%) and noninflammatory (68.3%) lesion counts after 12 weeks [20]. The reductions seen in inflammatory (78.3%) and noninflammatory (79.5%) lesion counts in the present study among Indian patients were greater than those reported earlier.

The difference in the extent of reduction in lesion counts could also be because of the difference in the number of baseline lesions in the reported studies, i.e., 87.5 total lesions in the present study compared with 39.8-67.2 [12,13] and 98.5 [21] in the previous studies.

In terms of acne severity based on the IGA score, a substantial improvement was observed in transitioning from moderate-to-severe acne at baseline to clear and almost clear skin at the end of the study, confirming the treatment’s efficacy in improving overall acne severity. Most patients rated their skin as “clear” (28.0%) or “almost clear” (40.9%).

Satisfaction rates with A0.3%+BPO2.5% in acne management have been reported to be 71.6% at 12 weeks [20] and 90.1% at 24 weeks [12]. Similarly, in the present study, there was a 91% satisfaction rate with the A0.3%+BPO2.5% treatment at week 12.

The strength of the study is that it fills a gap in the Indian clinical data on the adapalene/BPO combination gel, offering insights into its safety and efficacy.

However, the relatively small sample size, short-term follow-up, and use of subjective questionnaires to score reductions in acne severity are weaknesses of this study. Therefore, extrapolating these results to the larger Indian population would require future studies with larger sample sizes that also include patients with different skin types found across India and objective scoring systems for measuring reductions in acne severity. Studies on the long-term (>12 weeks) effectiveness and safety of this combination in Indian patients are also required.

## Conclusions

The combination of A0.3%+BPO2.5% was evaluated for its safety and efficacy in Indian patients with moderate and severe acne vulgaris. Safety assessments, focusing on local tolerability and AEs, showed that the combination gel was well tolerated throughout the study duration. The proportion of patients reporting erythema and scaling increased by week two and thereafter reduced by the end of week 12 with extended treatment. The proportion of patients reporting dryness decreased by week two, increased by week eight, and again decreased by week 12 with extended treatment. The targeted 50% reduction in inflammatory and noninflammatory lesion counts was noted in 87.12% and 82.6% of patients, respectively, emphasizing the efficacy of the treatment across different lesion types. Most investigators rated their patients’ skin as clear and almost clear at the end of the study using the IGA scale, confirming the efficacy of the treatment in improving overall acne severity. Further, the GAI score as assessed by the investigators showed an improvement in patient symptoms at week 12. A substantial proportion of patients expressed high satisfaction with this treatment. The current findings support the use of a combination of A0.3%+BPO2.5% as a valuable option for acne management in Indian patients.

## Appendices

**Table 5:** Inclusion and exclusion criteria.

Inclusion criteria
Patients in the age group of 12–40 years
Patients with a clinical diagnosis of moderate-to-severe facial acne vulgaris, defined by the following: A minimum of 25–100 inflammatory lesions (papules and pustules). A minimum of 30–150 noninflammatory lesions (open and closed comedones) in total (excluding the nose). No more than two acne nodules (≥1 cm)
Female patients of childbearing potential must have a negative urine pregnancy test (UPT) at the baseline visit (visit 1)
Female patients of childbearing potential must practice an effective method of contraception during the clinical trial and at least one month after the last clinical trial treatment application: medical contraception (combined oral contraceptives (estrogens and progesterone) or implanted or injectable contraceptives with a stable dose for one month before clinical trial entry, bilateral tubal ligation, a hormonal intrauterine device inserted at least one month before clinical trial entry, strict abstinence (one month before trial entry and agrees to continue for the duration of the trial), condom with spermicide, vasectomized partner (for at least three months before clinical trial entry))
Females of nonchildbearing potential, e.g., premenses, postmenopausal (absence of menstrual bleeding for one year without any other medical reason), hysterectomy, or bilateral oophorectomy
Patients who have read, understood, and signed the approved Informed Consent Form (ICF) before any participation in the clinical trial
Patients under the age of 18 years who have signed an assent form to participate in the clinical trial and their parent(s) or legal representatives who have read and signed the ICF before any clinical trial-related procedure
Patients willing and able to comply with the trial protocol, specifically by adhering to the visit schedule, concomitant therapy prohibitions, and treatment requirements
Patients must be willing to be photographed
Patients (and parents/guardians if the patient is under 18 years of age) must be willing to consent to the same

Exclusion criteria
Patients with acne conglobata, acne fulminans, secondary acne (chloracne, drug-induced acne, etc.), nodulocystic acne, or acne requiring systemic treatment
Patients with a history of lupus, atopic dermatitis, perioral dermatitis, dermatomyositis, or rosacea on the face
Previous treatment failure with the A0.3%+BPO 2.5% gel
Patients with damaged facial skin (e.g., tattoos, cuts, skin abrasions, eczema, or sunburned skin)
Female patients who are pregnant, lactating, or planning a pregnancy during the trial. Patients with known impaired hepatic or renal function
The wash-out period for patients on topical treatment or procedures on the face should be as follows: topical treatments such as corticosteroids, antibiotics, benzoyl peroxide, azelaic acid, dapsone, hydroxy acids, zinc-containing treatments, antiseptics, and other anti‑inflammatory or acne treatments (e.g., salicylic acid treatments/transdermal contraceptives used for acne) - <2 weeks. Retinoids - <4 weeks. Cosmetic/aesthetic procedures on the face (e.g., comedone extraction, desquamating, or abrasive agents, adhesive cleansing strips) - <1 week. Wax epilation - <2 weeks. Photodynamic therapy - <6 weeks. Laser therapy, microdermabrasion, deep chemical peel, and plastic surgery for acne - <3 months
The wash-out period for patients on systemic treatment should be as follows: corticosteroids (excluding locally acting forms, such as inhaled, intrathecal, or dermal application, at a distance from the face), tetracyclines, and other antibiotics (except penicillin) - <1 month. Oral retinoids/isotretinoin, antiandrogens, cyproterone acetate, chlormadinone acetate - <6 months. Spironolactone/drospirenone (unless at a stable dose for at least three months for drospirenone) - <3 months. Immunomodulators - <3 months. Oral contraceptives/oral dapsone for acne - <1 month. Patients with active or chronic skin allergies
Patients with a known or suspected allergy to the investigational product
Patients who have used tanning booths or lamps or had excessive ultraviolet (UV) radiation exposure within one month before clinical trial entry or anticipate intensive UV exposure during the trial (mountain sports, sailing, sunbathing, etc.)
Patients at risk concerning precautions, warnings, and contraindications
Patients with active infections requiring administration of antibiotics
Patients with a beard or other facial hair that might interfere with trial assessments
Patients with acute/chronic diseases, uncontrolled systemic diseases, or a history of major medical or psychiatric conditions or surgical interventions that, in the opinion of the investigator, might put the patients at risk
Patients under guardianship, hospitalized patients in a public or private institution for reasons other than research, and patients deprived of their freedom
Patients who have participated in another investigational product or device research trial within 30 days before enrollment
Patients unable to communicate or cooperate with the investigator due to language problems, poor mental development, or impaired cerebral function
